# Enhancing of Surface Quality of FDM Moulded Materials through Hybrid Techniques

**DOI:** 10.3390/ma17174250

**Published:** 2024-08-28

**Authors:** Monika Jabłońska, Olga Łastowska

**Affiliations:** 1 Faculty of Mechanical and Electrical Engineering, Polish Naval Academy, Jana Śmidowicza 69, 81-127 Gdynia, Poland; 2 Faculty of Marine Engineering, Gdynia Maritime University, Morska 81-87, 81-225 Gdynia, Poland; o.lastowska@wm.umg.edu.pl

**Keywords:** additive manufacturing, 3D printing, hybrid techniques, turning, FDM moulded materials, surface quality, surface roughness, microstructure, dimensional accuracy, roundness

## Abstract

With the rapid advancement of 3D-printing technology, additive manufacturing using FDM extrusion has emerged as a prominent method in manufacturing. However, it encounters certain limitations, notably in surface quality and dimensional accuracy. Addressing issues related to stability and surface roughness necessitates the integration of 3D-printing technology with traditional machining, a strategy known as the hybrid technique. This paper presents a study of the surface geometric parameters and microstructure of plastic parts produced by FDM. Sleeve-shaped samples were 3D-printed from polyethylene terephthalate glycol material using variable layer heights of 0.1 mm and 0.2 mm and then subjected to the turning process with PVD-coated DCMT11T304 turning inserts using variable cutting parameters. The cutting depth was constant at 0.82 mm. Surface roughness values were correlated with the cutting tool feed rate and the printing layer height applied. The selected specimen’s microstructure was studied with a Zeiss EVO MA 15 scanning electron microscope. The roundness was measured with a Keyence VR-6200 3D optical profilometer. The research results confirmed that the additional application of turning, combined with a reduction in the feed rate (0.0506 mm/rev) and the height of the printed layer (0.1 mm), reduced the surface roughness of the sleeve (Ra = 1.94 μm) and increased its geometric accuracy.

## 1. Introduction

As a result of the rapid development of 3D-printing technology, the additive method through FDM extrusion has become one of the primary manufacturing methods [1]. This method enables the reproduction of the desired geometry of an element in a short time at low cost. The printing process begins by heating the nozzle and worktable to the operating temperature dedicated to the specific material. The filament is thereafter extruded, and a layer thereof is applied to the platform. Next, the platform is lowered and the next layer of filament is applied. The material hardens and fuses with the layer below after flowing out of the nozzle [2,3,4]. In the additive manufacturing process, elements are made by applying thin layers of filament, layer by layer. The key results of 3D printing are the surface quality of the printed part, the accuracy of reproduction, and durability.

The surface roughness of the printed elements varies considerably, depending on the settings of the printing parameters, the filament used, and environmental conditions. For instance increasing printing layer thickness during 3D printing increases surface roughness, while raising the material melting temperature promotes a better surface finish [5,6]. Srinivasan et al. studied infill density and infill pattern on the surface roughness of PETG parts. They reported that surface roughness decreases with increased infill density. The grid infill pattern has more surface roughness than cubic and triangular infill patterns [7]. Morphological defects in printed PETG products occur at higher print speeds [8]. Garcia et al. investigated the addition of carbon fibre to PETG polymers and unreinforced polymers. They found that incorporating carbon fibre in PETG had a negative effect on dimensional accuracy, flatness, and surface roughness [9]. Factors such as humidity, salt, temperature, and UV radiation caused changes in surface roughness, mechanical properties, and texture [10].

Monitoring of surface roughness enables the achievement of optimum mechanical properties, mapping accuracy, and technical and design requirements. This in turn avoids the need for rejecting an element due to non-compliance with the requirements [11,12].

The quality of the resulting surface of the elements produced by printing is a factor that determines their use in a variety of fields, from prototyping to mass production. To eliminate imperfections, reduce surface roughness, or adapt the shape of the part to technical requirements, the printed elements can be machined, e.g., using a turning process. The combination of 3D-printing technology and traditional machining is known as hybrid machining [13,14,15]. Adding a machining stage is a common solution for increasing the quality of manufactured elements. Nevertheless, the process employs several variables with a wide range of values, e.g., the selection of the turning tool, including the cutting insert, the selection of cutting parameters, and the definition of machining conditions [16]. These variables determine the achievement of the assumed values of the object’s nominal dimensions and the parameters of the geometric structure of the surface (roughness, waviness, form deviations). These properties affect the functionality of the turned surface, fatigue limit, wear resistance, corrosion resistance, lubrication, friction, and tightness of joints [17]. They are also an indispensable element in the field of tribology, as they enable the machining process carried out to be evaluated and the quality of the outer layer to be determined [18].

Hashmi et al. [19,20,21] pointed out the need for finishing when the functional requirements of a product are not met, for example, due to low surface roughness or dimensional inaccuracy. Such inaccuracies are due to a number of factors, including shrinkage phenomena, the angle of application (vertical walls are very close to the nominal dimension, whereas completely horizontal walls deviate by a few tenths of a millimetre), the layer thickness, the viscosity of the medium, the chord error, shrinkage, burrs in the support structure, and the type of filler.

Scientific articles include presentations and analyses of novel hybrid machines. For example, an arc welding module is combined with a CNC milling machine in a hybrid machine for the production of composite structures in [22]. In this process, the shape of the object is first created by weld deposition and then finished by subsequent machining close to the reference model. Arc welding has many advantages, including higher deposition rates, lower costs, shorter execution times, and safer operation. An assimilation trial of laser metal deposition, turning, and milling is presented in a hybrid multi-tasking machine tool in [23]. A combination of CNC machining and a selective laser melting process is presented in [24]. The type of hybrid production presented has been shown to improve efficiency and reduce costs.

Hur et al. proposed a new hybrid system that optimises the manufacturing process by using an appropriate planning process. The system used extracts all the shape features that can be machined by the CNC and analyses potential tool collisions and tool access to the part surface for machining. The system has been used on an Eclipse RP test bench. The developed process planning system retains the same level of automation as current SLA and FDM technologies [25].

Integration of milling and layer production on a single workstation with the same reference point and fixtures, focusing on toolpath generation, eliminating the problem of misalignment, and different control panels are discussed in [26,27,28].

Mehtedi et al. studied machinability, roughness, and burr formation after a milling operation using a PETG filament. They concluded that obtaining a smoother surface requires the use of lower feed rates and depths of cut, together with higher spindle speeds [29].

Pandey et al. found that the surface quality of printed parts was unsatisfactory due to the staircase effect and chords on the part, as well as parameters such as air gaps between layers, the width of the layer, or the infill pattern used. They developed a semi-empirical model to evaluate surface roughness. The HCM (hot cutter machine) method was used for finishing, achieving a surface roughness of 0.3 μm with an 87% confidence level [30]. The effect of staircasing and pearling during the FDM printing process was also discussed by Potnis et al. in their article [31]. These factors result in inadequate surface roughness and lower dimensional accuracy.

The roughness parameter is considered a key factor influencing the performance of machine components. It is also a factor that has an influence on the nucleation of fatigue cracks [32]. Surface roughness comprises surface irregularities caused mainly by the type of machining or manufacturing process of the workpiece. Measurements are taken on a given elementary section, the length of which depends on the required roughness parameter [33,34].

A theoretical model related to the prediction of surface roughness for an STL input file was developed by Daekeon et al. [35]. Based on the surface angle of the facet, the roughness is calculated and a roughness distribution curve is generated, taking into account the surface angle and layer thickness.

In both 3D-printing and turning operations, the material used and its properties, characteristics, and applicability to the mechanical industry play equally important roles. A polyethylene terephthalate glycol (PETG) polymer was selected for this study because of its properties of high mechanical strength, impact resistance, low material shrinkage, and ease of processing [36,37,38]. This thermoplastic material is used to manufacture housings and covers, connectors, cushioning elements, brackets, frames, and gears. To improve the geometric accuracy and achieve the desired surface quality of the elements, they are sometimes shaped using machining. The machinability of PETG material depends on the type of process and mechanical properties [39]. No recommended cutting parameters for PETG material were found in the literature. As the glass transition temperature of PETG is 75 °C, a cutting speed of 30 m/min, turning depth of 0.82 mm, and feed rate of 0.05 ÷ 0.22 mm/rev were applied to prevent excessive heating of the workpiece. The main problem in machining these materials is the plastic turnings wrapped around the workpiece and the phenomenon of stringing [40].

The production of machine and equipment components that work together is increasingly achieved through 3D-printing technology, prompting the need to obtain these components in satisfactory condition in terms of wear and tear, durability, and economy.

The integration of various manufacturing techniques, including 3D-printing and turning processes, is one of the principles of Industry 4.0 strategy [32,41,42]. Combining multiple specialised fields enables the connection of these techniques and facilitates interdisciplinary navigation within the realm of the production process. The addressed topic can be applied in manufacturing through an automated quality control system integrated with a 3D printer. On the other hand, the widespread implementation of additive manufacturing in industry faces significant constraints related to achievable production quality. One potential solution is embracing hybrid manufacturing methods that incorporate additional processes such as machining [43]. This study aimed to determine the influence of printing and machining parameters on the obtainability of a specific surface roughness of an element made of PETG material and local roundness deviation.

The article is divided into the following sections. Section 1 provides a literature review and a discussion of the basic definitions and parameters related to hybrid processing. Section 2 describes the materials and test algorithms used (test specimen preparation algorithm, printing parameters, test methodology, description of the chosen filament, description of the test bench). Section 3 presents the test results and their analysis. The last section contains a discussion and the authors’ intentions for future research.

An important aspect of the research is the use of an innovative hybrid technique using PETG material, which has not been studied before and can contribute to an improved manufacturing process. Furthermore, this study can be used as a basis for further experiments on the selection of materials, parameters, and tools, due to the reproducible methodology and easily comparable measurement methods.

## 2. Materials and Methods

The following section elaborates the experimental procedure carried out, and the steps involved are: filament material, object of the study, fused deposition modelling, and testing algorithm.

### 2.1. Filament Material

Polyethylene terephthalate glycol (PETG) (ROSA PLAST SP. z o.o., Hipolitów, Poland) was chosen for the manufacture of the specimens. This thermoplastic polymer is known for its low shrinkage, high strength, non-brittleness, and excellent layer adhesion. The properties of PETG material are as follows:

- Density of the filament (1.29 g/cm^3^);
- Izod notched impact strength (4.7 kJ/m^2^);
- Tensile strength (20 MPa);
- Modulus of elasticity in tension (2980 MPa).

The diameter of the 3D-printing filament used was 1.75 mm. The purchased filament was wound onto a spool and dried in a tumble dryer at 50 °C for 24 h prior to printing.

### 2.2. Object of the Study

**Figure 1:**
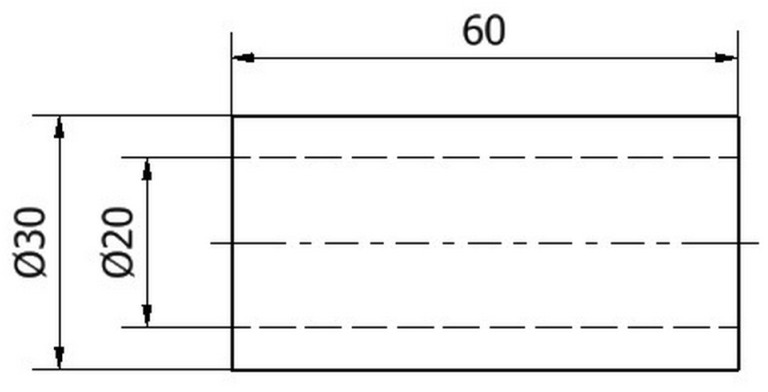
Model of the test specimen Ø30 × 5–60 mm.

The object of the study is a sleeve with dimensions of Ø30 × 5–60 mm, as shown in Figure 1 Machine parts class bushing is a difficult component to both print and machine on a lathe. During printing, there is little adhesion surface with the table and there is a risk that the printed part will detach from the surface of the work table. This will result in deformation of the part. During turning, vibration and oscillation may occur due to incorrect clamping of the part. Incorrect clamping can be caused by a misalignment of the centre of gravity of the sleeve, as in 3D printing, the walls can be unevenly distributed or have cavities in their structure. When the spindle starts to rotate and the workpiece has an offset centre of rotation, the centrifugal force increases, causing the workpiece to rotate in an eccentric manner. The result is an uneven cut.

### 2.3. Fused Deposition Modelling

**Figure 2:**
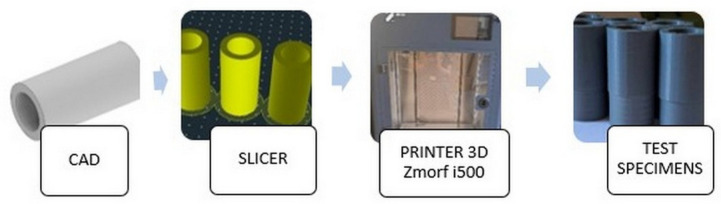
Algorithm for preparation of workpieces.

The sleeve was manufactured using the FDM incremental method on a Zmorf i500 (Zmorph S.A., Wrocław, Poland) industrial printer from a thermoplastic material in the form of PETG. The process was controlled by Slicer Voxelizer Industry software (Version: Voxelizer Industry 1.0.0, Zmorph S.A., Wrocław, Poland). Two types of samples were produced during the test. The variable value was the height of the printing layer and equalled 0.1 mm and 0.2 mm. Figure 2 shows the methodology for preparing the workpieces.

After printing, lines can be seen on the surface of the bushing. In cases where the bushing will act as a bearing, for example, this is undesirable, so a turning operation—a finishing operation—can be used to reduce the value of the surface roughness parameter.

The samples were printed as per the parameters shown in Table 1.

### 2.4. Testing Algorithm

The aim of the experimental study is to determine how the selected technological parameters, i.e., the height of the printing layer and the feed rate during turning, affect the surface roughness of the test piece. The interaction between the two is sought. Improving the surface quality of the printed part using the hybrid method has enabled the integration of manufacturing systems. The manufacturing process became flexible and competitive, allowing optimisation of the parameters used.

The experiment included a surface texture test carried out on printed parts of the bushing type.

**Figure 3:**
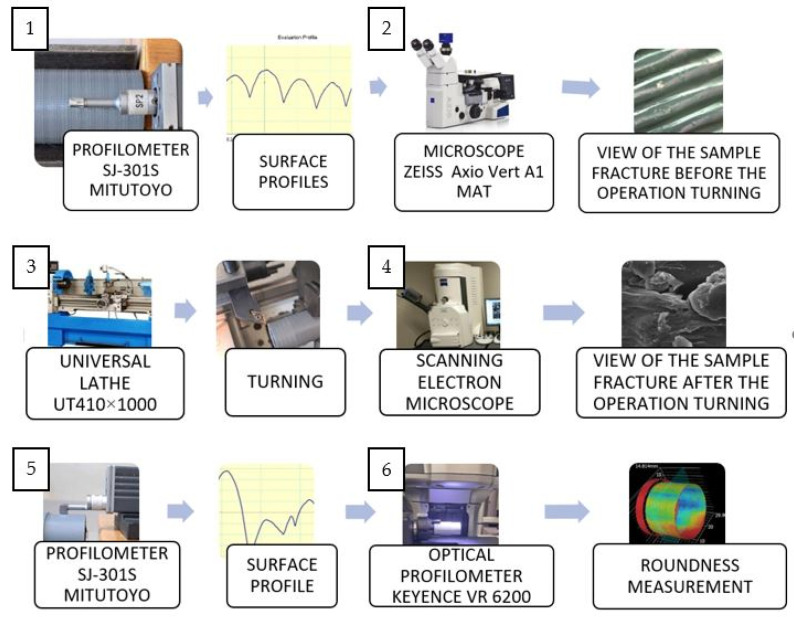
Testing algorithm.

The technological process used included the following operations, shown in Figure 3:

1. Testing the surface roughness of a test sample printed on a 3D printer using a Mitutoyo SJ-301S profilometer (Mitutoyo, Tokyo, Japan). Preparation of surface profiles related to the surface.
2. Examination of the state of the surface structure and its evaluation using a Zeiss Axio Vert A1 MAT microscope (Zeiss, Jena, Germany).
3. Longitudinal turning on a conventional lathe UT410 × 1000.
4. Examination of the surface structure and its evaluation using a scanning electron microscope.
5. Examination of the surface roughness of the specimen after turning using a Mitutoyo Pro-Filometer SJ-301S (Mitutoyo, Tokyo, Japan).
6. Roundness contour test with optical profilometer Keyence VR 6200 (KEYENCE, Itasca, IL, USA).

Turning technology was used to minimise the surface roughness values after the printing stage. Surface roughness tests of the samples, photography, and analyses of the microstructure of the sleeve surface was carried out after both the printing stage and the turning process. Modifications were made to variables such as the height of the printing layer and the feed value of the turning tool along the workpiece during machining.

#### 2.4.1. Surface Roughness Measurement of Printed Samples

**Figure 4:**
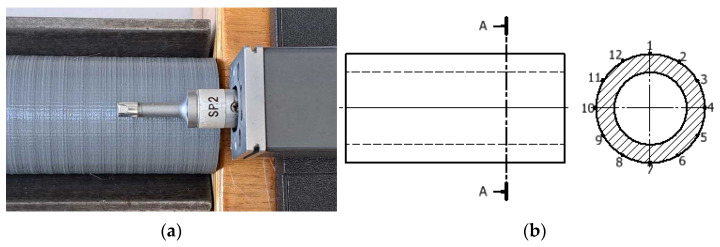
Roughness measurement scheme: (**a**) view of test bench—surface roughness measurement with profilometer, (**b**) location of measuring points on the sleeve.

A Mitutoyo SJ-301S contact profilometer was used to measure surface roughness (Figure 4). This instrument has a measuring range of 350 μm and uses a data acquisition system through a measuring tip placed in the head, which is in contact with the surface to be measured. The tip moves along the surface to be tested at a speed of 0.5 mm/s. Based on the initial roughness measurements, the elementary section lengths were selected for each specimen. Where Rz was in the range 0.5 ÷ 10 μm, the elementary section length was 0.8 mm, and in the range 10 ÷ 50 μm, it was 2.5 mm. The measurement uncertainty for Ra was ±0.083 μm and for Rz was ±0.28 μm.

The vertical movement of the profilometer tip resulting from surface irregularities was converted into a measurement signal of the position function. A total of twelve roughness measurements were carried out on the surface of each sample, each taken after rotating the sample by 30° around the axis of rotation.

#### 2.4.2. Microstructure

Based on the microscopic images obtained using the Zeiss Axio Vert A1 MAT microscope equipped with a PL10×/23 eyepiece, the surfaces of the samples were examined, referring to conditions before the machining step.

#### 2.4.3. Turning Process

**Figure 5:**
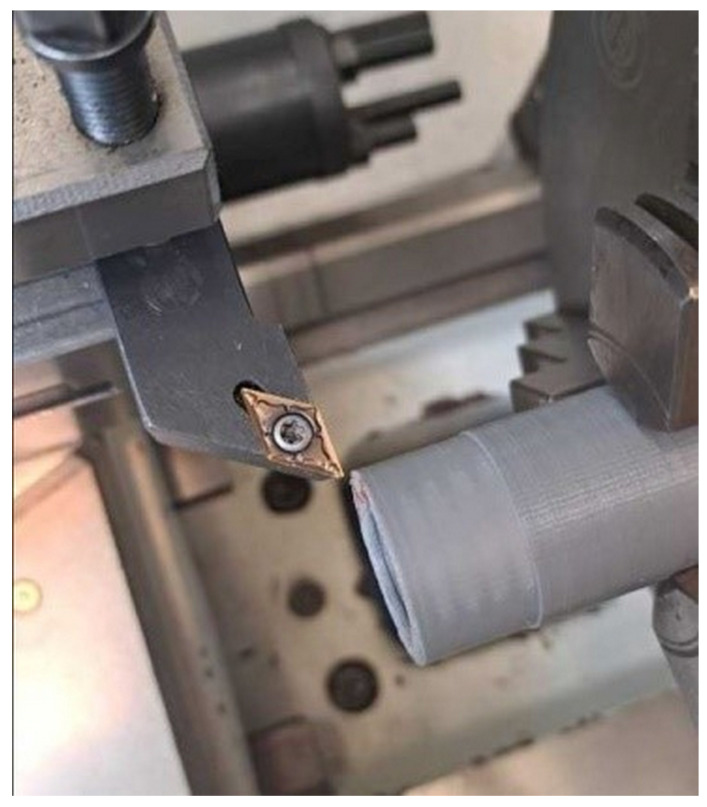
View of test bench—universal lathe UT 410 × 1000.

The test was conducted using a UT410 × 1000 horizontal axis universal lathe with a maximum spindle speed of 1800 rpm, maximum power of 3.3 kW, maximum turning diameter of 580 mm, and maximum turning length of 1000 mm [44]. Samples produced by the incremental method from PETG material with dimensions of Ø20/Ø30 × 60 mm were used for the study. The specimens were subjected to a dry longitudinal turning process using an SDJCR2020K11 folding lathe knife, using a turning insert with the DCMT11T304 symbol and an angle of 93. The turning insert had a main contact angle α = 7°, insert corner angle β = 55°, cutting-edge length 11.6 mm, and cutting-edge corner radius r_ε_ = 0.4 mm, and a chipbreaker F1 was used. The turning insert was a PVD-coated EM2520 carbide, with no chamfers on the cutting edges of the insert, ensuring the highest surface quality during machining. The technological process consisted of carrying out a longitudinal turning operation on a 20 mm section using the cutting parameters without the coolant supply attached. Figure 5 shows an example of the sample used in the study.

Difficulties with chip removal arose when turning the PETG material samples. The chips would wrap around the workpiece, which could lead to issues with machining accuracy and a lower surface roughness parameter value.

#### 2.4.4. Texture Testing

**Figure 6:**
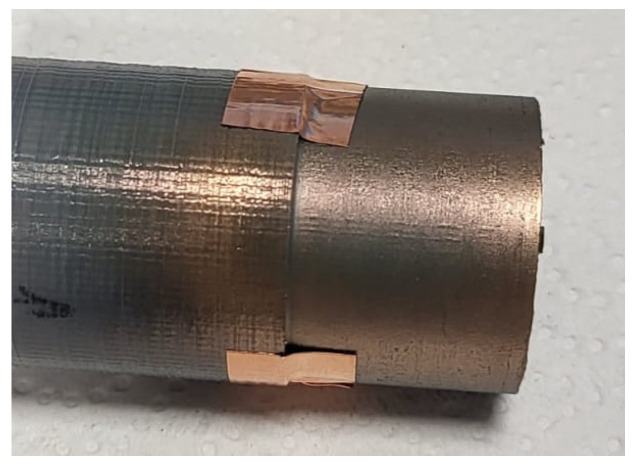
The sleeve, manufactured by FDM from PETG, was coated with gold.

Texture testing after the turning operation was performed using a Zeiss EVO MA 15 scanning electron microscope. This microscope is equipped with a backscattered electron detector BSD, secondary electron detector SE, and EDS analyser. The scanning was conducted in high-vacuum mode at an acceleration voltage of 20 kV and a vacuum pressure of 35 Pa [45]. The sample was coated with gold to achieve the desired magnification (Figure 6).

#### 2.4.5. Roundness Measurement of the Workpieces

**Figure 7:**
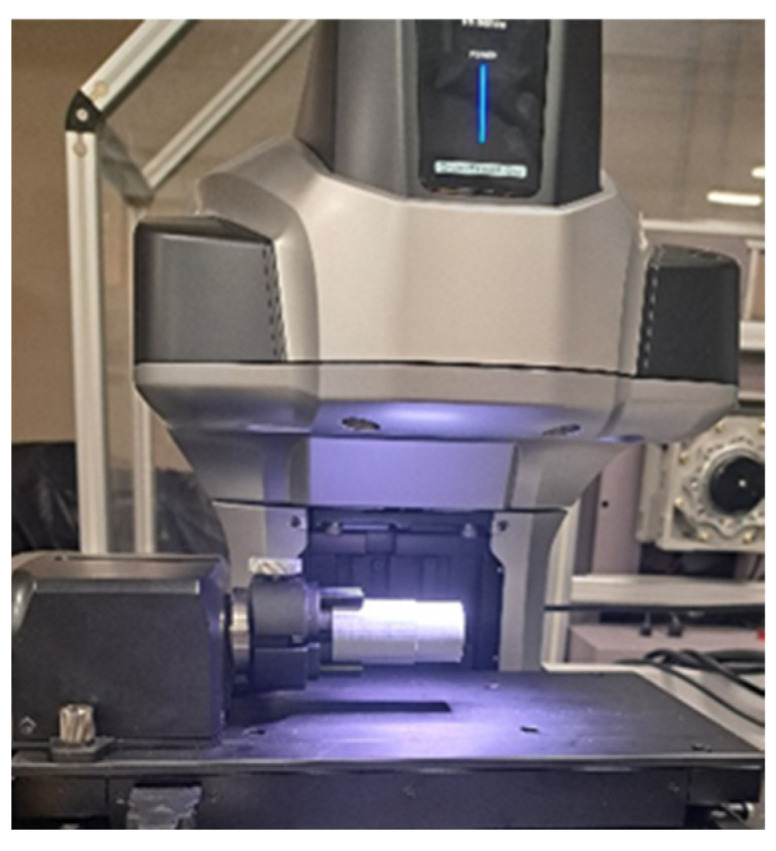
Keyence VR-6200 3D optical profilometer.

A VR-6200 series 3D optical profilometer was used for the study (Figure 7). This device is equipped with a monochrome CMOS sensor, 4 million pixels, dual telecentric lenses, LED ring, white LED, and rotating module with drive. It allows fully automatic measurement and has an automatic mapping function and area setting. The measurement accuracy for height is ±2.5 μm, for width ±2 μm. The VR-6200 optical profilometer used takes non-contact measurements [46,47].

## 3. Results and Discussion

The surface roughness values of the printed PETG samples were measured before turning for a printing layer height of 0.1 mm (36 measurements) and 0.2 mm (36 measurements). After the turning stage, further roughness measurements were taken for different printing layer heights *h* and different feed rates *fn* (0.1138 mm/rev, 0.0506 mm/rev and 0.2276 mm/rev, with the spindle speed *n* = 330 rpm and the cutting depth *a_p_* = 0.82 mm).

The surface microstructure of the printed part was examined using the Zeiss Axio Vert microscope. After finishing, the Zeiss EVO MA 15 scanning electron microscope was used for examination. The geometric deviation of the sleeve surface finish after machining was determined using the Keyence VR-6200 optical profilometer.

### 3.1. Surface Roughness Measurement of Printed Samples

Table 2 shows the results of the arithmetic mean of the 12 single-plane measurements obtained for the surface roughness parameters of the bushing after the 3D printing stage.

**Figure 8:**
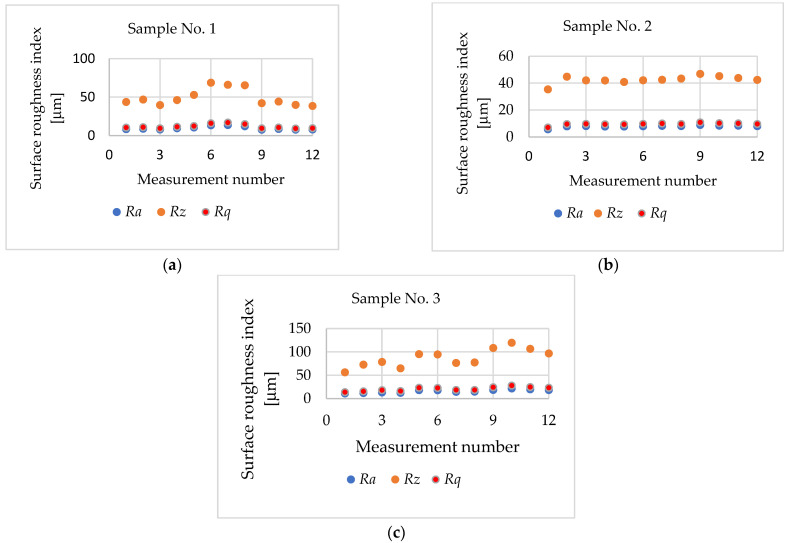
Surface roughness parameters of the sleeve after the 3D-printing stage, height of the printing layer h = 0.1 mm: (**a**) sample 1; (**b**) sample 2; (**c**) sample 3.

Figure 8 shows a graph of the surface roughness parameters of the PETG material sleeve while maintaining a printing layer height of 0.1 mm during printing. The highest value of the *Ra* roughness parameter was observed during the 10th measurement of sample 3 at 21.86 μm and the lowest value of 5.75 μm during the first measurement of sample 2. The *Rq* parameter, namely, mean squared deviation, was 28 μm and 7.08 μm, respectively. The highest value of the *Rz* parameter was obtained for sample 3, measurement 10, at 119.6 μm, while its lowest value was observed for sample 2, measurement 1, at 35.36 μm. The different values of the surface roughness parameters obtained, despite the application of convergent printing parameters, resulted from impurities present in the filament (Figure 7 and Figure 8) and the phenomenon of stringing [48].

**Figure 9:**
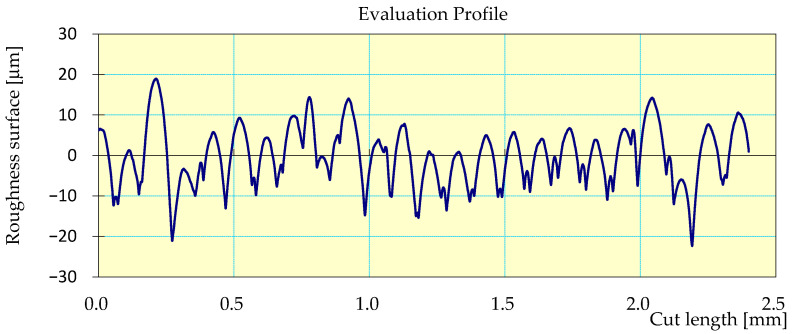
Profilogram, workpiece 2, measurement 1, printing layer height *h* = 0.1 mm, *Ra* = 5.75 μm; *Rz* = 35.36 μm, *Rq* = 7.08 μm, cut-off 2.5 mm, filter 2CR.

Figure 9 shows an example of the surface roughness profilogram of the workpiece from the second measurement, during which the lowest *Ra* value was obtained. Printing was carried out with a printing layer height of 0.1 mm.

### 3.2. Microstructure

**Figure 10:**
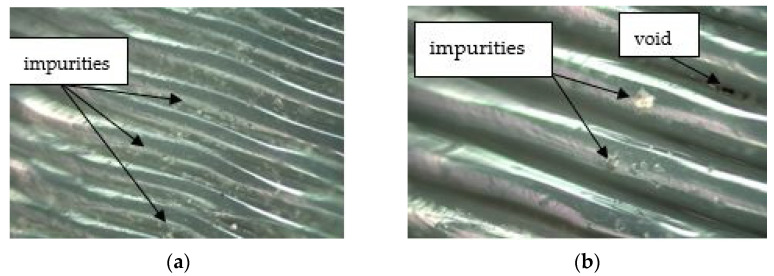
Microstructure of the surface of a sleeve manufactured by FDM from PETG: (**a**) workpiece 1, height of printing layer 0.1 mm, magnification × 100, gamma 2.0; (**b**) workpiece 1, height of printing layer 0.2 mm, magnification × 100, gamma 2.79.

Figure 10 shows successive layers of filament arranged in parallel and intermingling in places. Numerous impurities and inclusions were observed, which may be due to imperfections in the filament and may have been applied to the layer during printing. The impurities were visible as irregular, lighter, rusty areas on the surface of the filament.

### 3.3. Measurement of Surface Roughness after Turning

**Figure 11:**
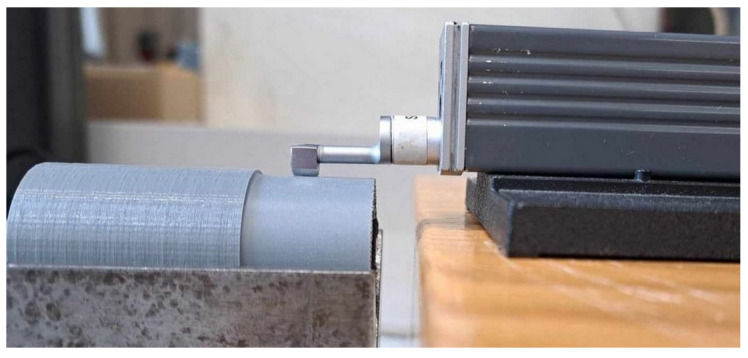
View of test bench—profilometer.

After the longitudinal turning stage, the surface roughness parameter was measured using the profilometer shown in Figure 11.

Table 3 shows the cutting parameters used in the longitudinal turning process, i.e., feed rate *f* (mm/rev), spindle speed *n* (rpm), and depth of cut *a_p_* (mm). The arithmetic mean values of the 12 roughness parameter measurements, standard deviation *σ* for the specified printing, coefficient of variation, and cutting process parameters are also included in the table. For a print height of 0.1 mm, the coefficient of variation was over 0.2, while for a print height of 0.2 mm, it was in the range of 0.1 ÷ 0.2. A higher coefficient of variation means a larger standard deviation from the mean, indicating a greater dispersion of the data around the mean.

**Figure 12:**
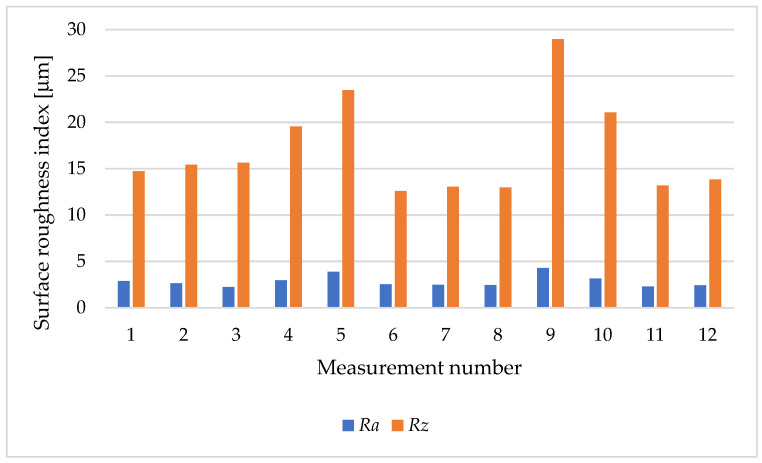
Roughness parameters *Ra* i *Rz*: sample 1, height of printing layer *h* = 0.1 mm, feed rate *fn* = 0.1138 mm/rev.

Figure 12 shows the values of the analysed surface roughness parameters of sleeve 1, in which a turning operation was performed on a 20 mm section with a 0.4 mm corner radius insert.

**Figure 13:**
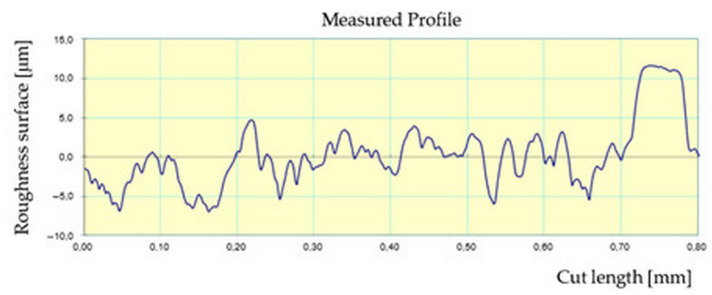
Workpiece 1 profilogram: printing layer height *h* = 0.1 mm, *Ra* = 1.96 μm; *Rz* = 13.58 μm, *Rq* = 2.42 μm, cut-off 0.8 mm, filter 2CR, feed rate *fn* = 0.0506 mm/rev.

Figure 13 shows a surface roughness profilogram of the test specimen, measurement 1, after the machining process stage. The printing was carried out with a printing layer height of 0.1 mm.

**Figure 14:**
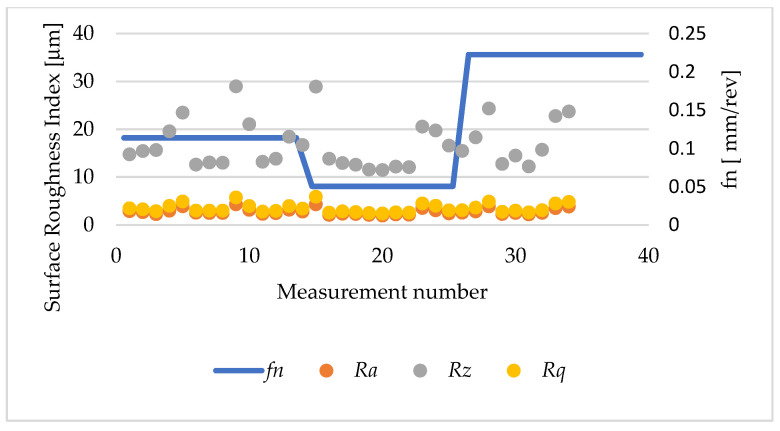
Surface roughness parameters of the sleeve after cutting with the use of DCMT11T304. Insert: height of the printing layer *h* = 0.1 mm.

Figure 14 shows the values of the surface roughness parameters of the sleeve after the turning operation with the DCMT117304 insert. The lowest value of the roughness parameter *Ra* of 1.94 μm was obtained in sample 2, measurement 20, which used the lowest feed rate *f_n_* of 0.0506 mm/rev and a depth of cut layer of 0.82 mm. An increase in *Ra* to 4.28 μm occurred on the surface of sample 1, measurement 9, for the following cutting parameters: *f_n_* equal to 0.1138 mm/rev, *a_p_* equal to 0.82 mm, and *n* equal to 330 rpm.

**Figure 15:**
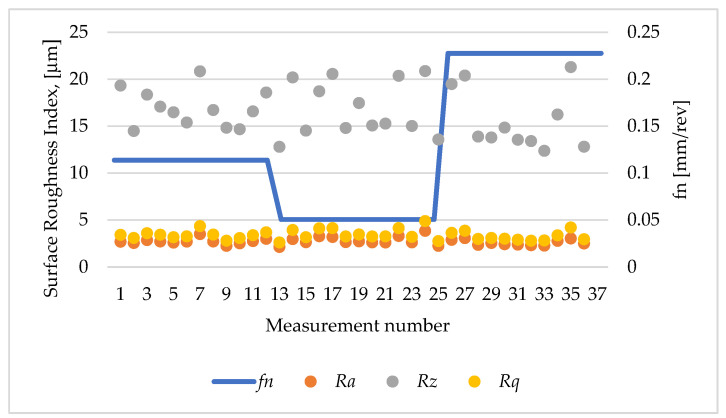
Surface roughness parameters of the sleeve after the cutting with DCMT11T304. Insert: height of the printing layer *h =* 0.2 mm.

Figure 15 shows a graph of the effect of the cutting tool feed rate on the value of the roughness parameter. The results obtained for the *Ra* parameter ranged from 2.13 μm to 3.5 μm. The smallest value of the *Ra* parameter was obtained with a feed rate *fn* equal to 0.0506 mm/rev (sample 2, measurement 1), and the highest for *fn* equal to 0.1138 mm/rev (sample 1, measurement 7).

### 3.4. Texture Testing

**Figure 16:**
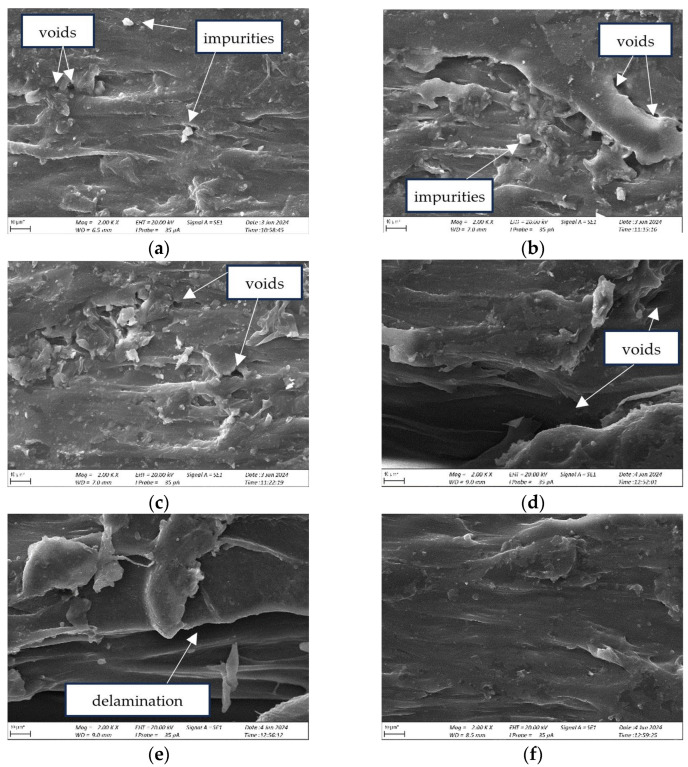
Microstructure of the surface of a sleeve manufactured by FDM from PETG: (**a**) workpiece 1, height of printing layer 0.1 mm, magnification × 2000, *fn* = 0.1138 mm/rev, *n* = 330 rpm, *a_p_* = 0.82 mm; (**b**) workpiece 2, height of printing layer 0.1 mm, magnification × 2000, *fn* = 0.0506 mm/rev, *n* = 330 rpm, a_p_ = 0.82 mm; (**c**) workpiece 3, height of printing layer 0.1 mm, magnification × 2000, *fn* = 0.2276 mm/rev, *n* = 330 rpm, *a_p_* = 0.82 mm; (**d**) workpiece 4, height of printing layer 0.2 mm, magnification × 2000, *fn* = 0.1138 mm/rev, *n* = 330 rpm, *a_p_* = 0.82 mm; (**e**) workpiece 5, height of printing layer 0.2 mm, magnification × 2000, *fn* = 0.0506 mm/rev, *n* = 330 rpm, *a_p_* = 0.82 mm; (**f**) workpiece 6, height of printing layer 0.2 mm, magnification × 2000, *fn* = 0.2276 mm/rev, *n* = 330 rpm, *a_p_* = 0.82 mm.

Figure 16 shows the microstructures of the sleeve surfaces after the turning. The SEM images show the structural changes and damage. Observations included an irregular surface, cracks, displacements, and bond deformation [49,50]. Voids located at the interface between individual layers on the same printing plane are visible.

In Figure 16a, it was observed that at a feed rate of 0.1138 mm/rev and a printing layer height of 0.1 mm, microcracks, small voids, and a peeling-like structure were formed in the material. Similar defects appeared at feed rates of 0.0506 mm/rev and 0.2276 mm/rev, where small voids and fine debris were also visible. When printing the part with a layer height of 0.2 mm after the turning stage, the use of feed rates of 0.1138 mm/rev and 0.0506 mm/rev resulted in delamination and voids. The least material degradation was observed in specimen 6, where only minor delamination occurred. In this case, the parameters were 0.2276 mm/rev for the feed rate and 0.2 mm for the printing layer height.

### 3.5. Roundness Measurement of the Workpieces

**Figure 17:**
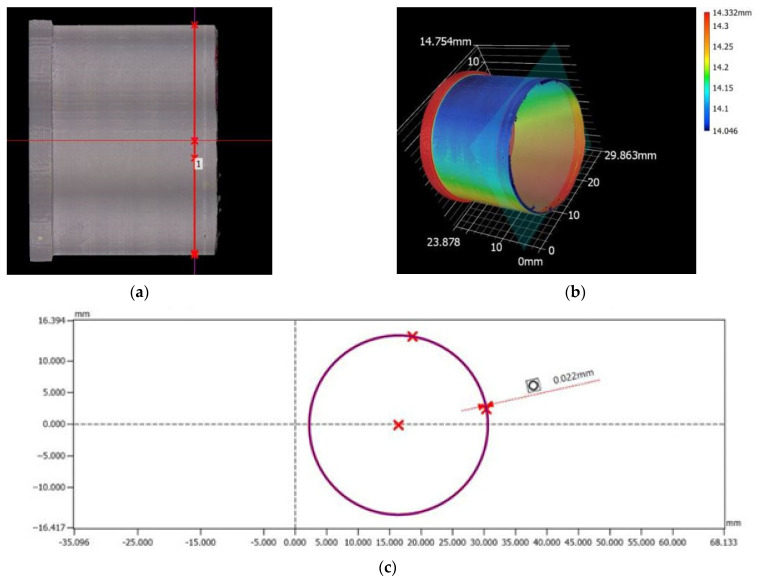
Profile measurement. Specimen 2: (**a**,**b**) distance of the plane of roundness from the face of the specimen; (**c**) local roundness deviation.

The circumferential line on each cross section of the cylindrical surface was analysed during the tests. The roundness plane was applied three times to each specimen, at distances of 4 mm, 10 mm, and 16 mm from the specimen face (Figure 17). Table 4 shows the results of the local roundness deviation. The smallest deviation in roundness was shown in specimen 2 and was 0.022 mm. The narrowest deviation was found in specimen 6 and was 0.07 mm.

The inhomogeneity of the material, the thermal deformation occurring, the vibrations, and the parameters used during turning influence the magnitude of the roundness deviations and the dimensional accuracy [50].

Specimen 2 had a printing layer height of 0.1 mm and the feed rate used was 0.0506 mm/rev. Specimen 6 had a printing layer height of 0.2 mm and the feed rate was 0.2276 mm/rev.

The results of the developed methodology mean that it is possible to determine the interdependence between the height of the print layer, the feed speed of the cutting tool, and the parameter of surface roughness obtained. Relationships were developed for a cylindrical component printed using a 3D printer by extrusion from PETG material. A filament commonly used to print mechanical parts was chosen for the study, and a conventional machine tool was used for finishing. Both of these pieces enabled the transfer of the results of experimental tests to operational conditions because of their universality.

## 4. Conclusions

**Figure 18:**
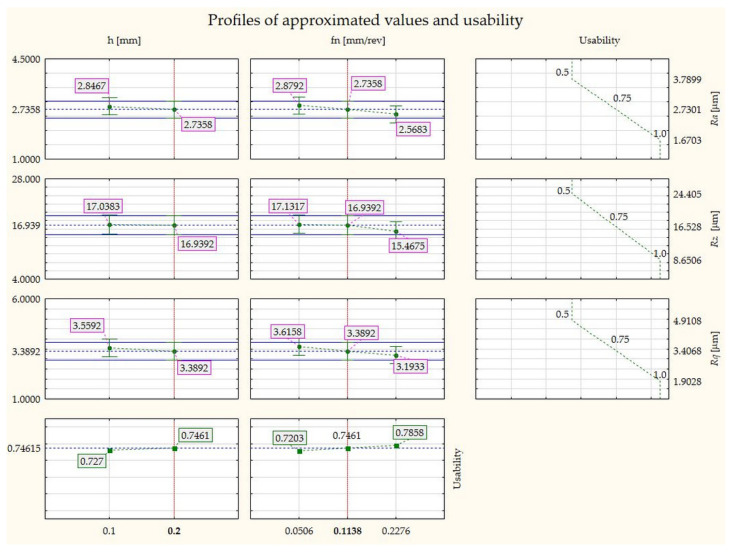
ANOVA plots. Comparison of profiles of approximated values for printing layer height, feed rate, and usability.

This study focused on optimising the parameters of the hybrid process, i.e., the height of the printing layer and the feed rate during the turning process for the PETG material. The surface roughness before and after turning, the condition of the surface structure, and the contour of the roundness were investigated. A usability analysis based on the results of the study made it possible to determine the relationship between the variables: surface roughness *Ra, Rz, Rq*, feed rate *fn,* printing layer height, and usability. The plot of the results is shown below (Figure 18).

The *Ra* value remains stable over the whole range of h values, with slight fluctuations around an average of 2.7358 μm. The *Ra* value also remains stable over the fn range, fluctuating from approximately 2.5683 μm to 2.8792 μm. It has been assumed that the utility decreases as *Ra* increases. When Ra is 1.6703 μm, the utility is 1. When Ra is 2.7301 μm, the utility is 0.75, and when Ra is 3.7899 μm, the utility is 0.5.

From a usability perspective, lower roughness values are more desirable.

Utility is fairly stable over the range of h values, varying from approximately 0.727 to 0.7461. Utility is also stable across the range of fn values, varying from approximately 0.7203 to 0.7858.

1. The research programme carried out allowed detailed conclusions to be drawn.
2. Samples printed at a layer height of 0.1 mm had lower roughness parameters compared to samples for which a layer height of 0.2 mm was used. These amounted to *Ra* = 7.95 μm and *Ra* = 12.94 μm, respectively.
3. The hybrid technology developed enabled a component surface parameter for a 0.1 *Ra* printing layer height of 2.52 μm, and for a 0.2 mm *Ra* layer height of 2.56 μm to be obtained.
4. Comparing the surface microstructure of the element with 0.1 mm and 0.2 mm layer heights, it can be observed that better bonding of the printing layers occurred with the samples where a 0.1 mm height was used. Greater adhesion between the layers also translated into a more accurate representation of the model geometry and a lower surface roughness parameter.
5. The results confirmed that the surface roughness parameter decreases as the tool feed rate decreases. Using a layer height of 0.1 mm and a feed rate of 0.0506 mm/rev, the *Ra* parameter was 1.94 μm, at 0.1138 mm/rev, it was 2.22 μm, and at 0.2226 mm/rev, it was equal to 2.39 μm. Maintaining the height of the 0.2 mm printing layer, the *Ra* parameter was 2.13 μm for a feed rate of 0.0506 mm/rev, 2.25 μm for 0.1138 mm/rev, and 2.25 μm for 0.2226 mm/rev.
6. Specimen 2, with a lower printing layer height (0.1 mm) and lower feed rate (0.0506 mm/rev), showed the smallest roundness deviation of 0.022 mm. In contrast, specimen 6, with a higher printing layer height (0.2 mm) and higher feed rate (0.2276 mm/rev), had a higher roundness deviation of 0.07 mm. Increasing the height of the printing layer during printing and using a higher feed rate during turning can lead to larger roundness deviations. This may be due to higher cutting forces and vibrations.

The use of hybrid technology allowed the integration of manufacturing processes, i.e., 3D printing and turning: 3D printing ensured short production times and minimised waste, while turning compensated for imperfections and dimensional differences. The combination of the two processes provided greater versatility and a wider range of options.

The use of hybrid techniques allows greater design flexibility and integration of manufacturing processes, in this case, 3D printing and turning. The 3D printing enables rapid production, waste minimisation, and a wide range of materials. Machining on a lathe compensates for imperfections and dimensional variations. The combination of these two methods provides greater versatility and expanded possibilities.

The research was limited by the test and production equipment used. A multi-purpose lathe was selected with the ability to adjust the spindle speed to 16 different positions, from 45 rpm to 1800 rpm. The longitudinal feed range was 0.05 to 1.7 mm/rev. A CNC lathe with infinitely variable spindle speed and feed is planned for further testing. During printing, the minimum adjustable layer height was 0.1 mm, so the finishing operation was carried out on the lathe to improve the surface finish.

Further research will focus on evaluating the influence of various combinations, i.e., printing speed, spindle speed, print layer height, depth of cut, and extrusion temperature, on surface roughness.

## Figures and Tables

**Table 1:** 3D-printing parameters used in printing workpieces.

Parameter	Value
Filament	PETG
Extruder temperature maintained during printer operation	225 °C
Temperature of the table for all extruded printing layers	70 °C
Infill density of the sample	100%
Number of outer layers of the printed object	4
Printing layer height	0.1 and 0.2 mm
First printing layer height	0.25 mm
First printing layer width	0.4 mm
Infill pattern inside the sample	linear
Feed rate during which the filament layer is extruded	30 mm/s
Infill angle	45°
Infill type	rectilinear

**Table 2:** Results of sleeve surface roughness measurements after the printing stage: *h*—printing layer height; $\bar{X}$—arithmetic mean of 12 measurements; σ—standard deviation.

Sample	*h* (mm)	*Ra* (μm)		*Rz* (μm)		*Rq* (μm)	
		$\bar{X}$	σ	$\bar{X}$	σ	$\bar{X}$	**σ**
1	0.1	9.70	2.22	49.51	11.07	11.80	2.70
2	0.1	7.95	0.76	42.64	2.84	9.65	0.90
3	0.1	16.09	3.5	87.33	19.19	20.68	4.36
4	0.2	13.55	1.82	65.75	8.89	16.48	2.38
5	0.2	12.94	1.00	62.21	5.33	15.65	1.20
6	0.2	12.85	0.71	64.44	3.92	15.70	0.96

**Table 3:** Technological parameters for cutting, printing and surface roughness parameters after turning. Summary of arithmetic means (from 12 measurements/samples), standard deviation, and coefficient of variation.

Sample	*h* (mm)	*f_n_* (mm/rev)	*Ra* (μm)			*Rz* (μm)		
			$\bar{X}$	*σ*	CV	$\bar{X}$	*σ*	CV
1	0.1	0.1138	2.85	0.64	0.22	17.03	5.15	0.30
2	0.1	0.0506	2.52	0.72	0.28	15.05	5.37	0.35
3	0.1	0.2276	2.90	0.61	0.21	18.04	4.17	0.23
4	0.2	0.1138	2.74	0.30	0.11	16.94	2.00	0.11
5	0.2	0.0506	2.88	0.45	0.15	17.13	2.88	0.16
6	0.2	0.2276	2.56	0.29	0.11	15.47	3.14	0.20

**Table 4:** Local roundness deviation (mm).

Distance from the Plane of Roundness to the Face of the Specimen [mm]	Roundness Deviation Specimen					
	1	2	3	4	5	6
16	0.034	0.033	0.035	0.032	0.028	0.033
10	0.029	0.049	0.055	0.03	0.038	0.07
4	0.045	0.022	0.048	0.025	0.05	0.03

## Data Availability

The data presented in this study are available on request from the corresponding author. The data are not publicly available due to privacy restrictions.

## References

[1] (2015). Additive Manufacturing—General Principles—Terminology.

[2] Jabłońska M., Jurczak W., Ozimina D., Adamiak M. (2023). Increasing the operational reliability of a ship by using a composite impeller in the event of hydrophore pump failure. Eksploat. Niezawodn.—Maint. Reliab..

[3] Górski F., Wichniarek R., Kuczko W., Zawadzki P., Buń P. (2015). Strength of ABS parts produced by fused deposition modelling technology—A critical orientation problem. Adv. Sci. Technol. Res. J..

[4] Nagaraju D.S., Krupakaran R.L., Sripadh C., Nitin G., Joy Joseph Emmanuel G. (2023). Mechanical properties of 3D printed specimen using FDM (Fused deposition modelling) and SLA (Stereolithography) technologies. Mater. Today Proc..

[5] Chaidas D., Kitsakis K., Kechagias J., Maropoulos S. (2016). The impact of temperature changing on surface roughness of FFF process. IOP Conf. Ser. Mater. Sci. Eng..

[6] Kovan V., Tezel T., Topal E., Camurlu H. (2018). Printing Parameters Effect on Surface Characteristics of 3D Printed Pla Materials. Int. Sci. J. Mach. Technol. Mater..

[7] Srinivasan R., Prathap P., Raj A., Aswinth Kannan S., Deepak V. (2020). Influence of fused deposition modeling process parameters on the mechanical properties of PETG parts. Mater. Today Proc..

[8] Loskot J., Jezbera D., Loskot R., Bušovský D., Barylski A., Glowka K., Duda P., Aniołek K., Voglová K., Zubko M. (2023). Influence of print speed on the microstructure, morphology, and mechanical properties of 3D-printed PETG products. Polym. Test..

[9] García E., Núñez P.J., Caminero M.A., Chacón J.M., Kamarthi S. (2022). Effects of carbon fibre reinforcement on the geometric properties of PETG-based filament using FFF additive manufacturing. Compos. Part B Eng..

[10] Głowacki M., Mazurkiewicz A., Słomion M., Skórczewska K. (2022). Resistance of 3D-Printed Components, Test Specimens and Products to Work under Environmental Conditions—Review. Materials.

[11] Ciecieląg K. (2023). Analysis of the Surface Layer and Feed Force after Milling Polymer Composites with Coated and Uncoated Tools. Adv. Sci. Technol. Res. J..

[12] Al-Sabur R., Kubit A., Khalaf H.I., Jurczak W., Dzierwa A., Korzeniowski M. (2023). Analysis of Surface Texture and Roughness in Composites Stiffening Ribs Formed by SPIF Process. Materials.

[13] Mertkan İ.A., Tezel T., Kovan V. (2023). Improving surface and dimensional quality with an additive manufacturing-based hybrid technique. Int. J. Adv. Manuf. Technol..

[14] Djurović S., Lazarević D., Mišić M., Šarkoćević Ž., Golubović Z., Mitrovic N., Mladenovic G., Mitrovic A. (2024). 3D Printing and CNC Machining: Materials, Technologies, and Process Parameters. New Trends in Engineering Research.

[15] Tejada Martinez L.V., Witz J.-F., Najjar D., Boidin X., Lesaffre F., Martin V., Badin S., Berte E. (2024). Hybrid FFF/CNC: An open source hardware & software system. HardwareX.

[16] Du Y., Mukherjee T., Finch N., De A., DebRoy T. (2022). High-throughput screening of surface roughness during additive manufacturing. J. Manuf. Process..

[17] He C.L., Zong W.J., Zhang J.J. (2018). Influencing factors and theoretical modeling methods of surface roughness in turning process: State-of-the-art. Int. J. Mach. Tools Manuf..

[18] Miko E., Nowakowski Ł. (2012). Analysis and Verification of Surface Roughness Constitution Model after Machining Process. Procedia Eng..

[19] Boschetto A., Bottini L., Veniali F. (2016). Finishing of Fused Deposition Modeling parts by CNC machining. Robot. Comput.-Integr. Manuf..

[20] Wahab Hashmi A., Ahmad S., Gulam Mustafa M., Tian Y., Iqbal F., Singh Mali H., Kamyab H., Yusuf M. (2024). Abrasive flow finishing of 3D-Printed Aerofoils: Design, numerical Simulation, and experimental analysis. Opt. Laser Technol..

[21] Taufik M., Jain P.K. (2017). Laser assisted finishing process for improved surface finish of fused deposition modelled parts. J. Manuf. Process..

[22] Karunakaran K.P., Suryakumar S., Pushpa V., Akula S. (2010). Low cost integration of additive and subtractive processes for hybrid layered manufacturing. Robot. Comput.-Integr. Manuf..

[23] Yamazaki T. (2016). Development of A Hybrid Multi-tasking Machine Tool: Integration of Additive Manufacturing Technology with CNC Machining. Procedia CIRP.

[24] Li Z., Yang Z., Liu B., Yang S., Kuai Z., Li J., Li H., Chen Y., Wu H., Bai P. (2022). Microstructure and mechanical properties of CNC-SLM hybrid manufacturing 316L parts. J. Manuf. Process..

[25] Hur J., Lee K., Zhu-hu Kim J. (2002). Hybrid rapid prototyping system using machining and deposition. Comput.-Aided Des..

[26] Kulkarni P., Dutta D. (1999). On the Integration of Layered Manufacturing and Material Removal Processes. J. Manuf. Sci. Eng..

[27] Amanullah A., Murshiduzzaman, Saleh T., Khan R. (2017). Design and Development of a Hybrid Machine Combining Rapid Prototyping and CNC Milling Operation. Procedia Eng..

[28] Lee W., Wei C., Chung S.-C. (2014). Development of a hybrid rapid prototyping system using low-cost fused deposition modeling and five-axis machining. J. Mater. Process. Technol..

[29] Mehtedi M.E., Buonadonna P., Mohtadi R.E., Aymerich F., Carta M. (2024). Surface quality related to machining parameters in 3D-printed PETG components. Procedia Comput. Sci..

[30] Pandey P.M., Venkata Reddy N., Dhande S.G. (2003). Improvement of surface finish by staircase machining in fused deposition modeling. J. Mater. Process. Technol..

[31] Potnis M.S., Singh A., Jatti V.S., Sapre M.S., Pathak S., Joshi S., Jatti A.V. (2024). Part quality investigation in fused deposition modelling using machine learning classifiers. Int. J. Interact. Des. Manuf..

[32] Grzesik W. Influence of Surface Roughness on Fatigue Life of Machine Elements—The Development in Experimental Investigations and Simulations. In Mechanik Miesięcznik Naukowo-Techniczny. https://www.mechanik.media.pl/artykuly/wplyw-chropowatosci-powierzchni-na-trwalosc-zmeczeniowa-elementow-maszyn-postep-w-dziedzinie-badan-i-symulacji.html.

[33] Gadelmawla E.S., Koura M.M., Maksoud T.M.A., Elewa I.M., Soliman H.H. (2002). Roughness parameters. J. Mater. Process. Technol..

[34] (2022). Geometrical Product Specifications (GPS)—Surface Texture: Profile—Part 2: Terms, Definitions and Surface Texture Parameters.

[35] Ahn D., Kim H., Lee S. (2009). Surface roughness prediction using measured data and interpolation in layered manufacturing. J. Mater. Process. Technol..

[36] Zubrzycki J., Estrada Q., Staniszewski M., Marchewka M. (2022). Influence of 3D Printing Parameters by FDM Method on the Mechanical Properties of Manufactured Parts. Adv. Sci. Technol. Res. J..

[37] Tamrin K.F., Nukman Y., Sheikh N.A. (2015). Laser Spot Welding of Thermoplastic and Ceramic: An Experimental Investigation. Mater. Manuf. Process..

[38] Mahesh V., George J.P., Mahesh V., Chakraborthy H., Mukunda S., Ponnusami S.A. (2023). Dry-sliding wear properties of 3D printed PETG/SCF/OMMT nanocomposites: Experimentation and model predictions using artificial neural network. J. Reinf. Plast. Compos..

[39] Ajay Kumar M., Khan M.S., Mishra S.B. (2020). Effect of machine parameters on strength and hardness of FDM printed carbon fiber reinforced PETG thermoplastics. Mater. Today Proc..

[40] Zaleski K., Matuszak J. (2017). Comparative study of the influence of technological parameters of milling of selected titanium alloys on cutting torque and surface roughness of machined surface. Adv. Mech. Mater. Eng..

[41] Prashar G., Vasudev H., Bhuddhi D. (2023). Additive manufacturing: Expanding 3D printing horizon in industry 4.0. Int. J. Interact. Des. Manuf..

[42] Görçün Ö.F., Mishra A.R., Aytekin A., Simic V., Korucuk S. (2024). Evaluation of Industry 4.0 strategies for digital transformation in the automotive manufacturing industry using an integrated fuzzy decision-making model. J. Manuf. Syst..

[43] Dilberoglu U.M., Gharehpapagh B., Yaman U., Dolen M. (2021). Current trends and research opportunities in hybrid additive manufacturing. Int. J. Adv. Manuf. Technol..

[44] (2018). Lathe Operator’s Manual UT 410x1000. https://www.cormak.pl/gb/universal-lathes/263-410x1000-universal-lathe-5906800305619.html?srsltid=AfmBOorHx0NQfYfD0a0JlJRQpc6rVITBGUnJrqhbM6yzwppJ0a76pfEV.

[45] Sepahi M.T., Abusalma H., Jovanovic V., Eisazadeh H. (2021). Mechanical Properties of 3D-Printed Parts Made of Polyethylene Terephthalate Glycol. J. Mater. Eng. Perform..

[46] Marshal R.M., Patzek M., Rüsch O. (2024). Characterization of the micrometer scale surface roughness of meteoritic samples. Icarus.

[47] Gapiński B. (2007). Recommendations for the roundness measurement with, CMM. Pomiary Autom. Kontrola R.

[48] Labuda W., Dargacz M., Klecha M., Kozłowska S. (2015). The influence of changing the side angle of the cutting tool by wiper technology on the value of surface roughness parameters of shaft pins made of austenitic steel. Sci. J. Gdyn. Marit. Univ..

[49] Lakshman Sri S.V., Karthick A., Dinesh C. (2024). Evaluation of mechanical properties of 3D printed PETG and Polyamide (6) polymers. Chem. Phys. Impact.

[50] de Menezes E.A.W., Friedrich L., Colpo A., Amico S.C., Thomas S., Hosur M., Chirayil C.J. (2019). Chapter 5—Micromechanics of Short-Fiber and Particulate Composites. Unsaturated Polyester Resins.

